# Antioxidant activity and protection against oxidative-induced damage of *Acacia shaffneri* and *Acacia farnesiana* pods extracts: in vitro and in vivo assays

**DOI:** 10.1186/s12906-015-0959-y

**Published:** 2015-12-15

**Authors:** Claudia Delgadillo Puga, Mario Cuchillo Hilario, José Guillermo Espinosa Mendoza, Omar Medina Campos, Eduardo Molina Jijón, Margarita Díaz Martínez, Marsela Alejandra Álvarez Izazaga, José Ángel Ledesma Solano, José Pedraza Chaverri

**Affiliations:** National Institute of Medical Science and Nutrition “Salvador Zubirán”, Vasco de Quiroga 15., Mexico City, 14000 Mexico; Department of Biology, Faculty of Chemistry, National Autonomous University of Mexico, Mexico City, 04510 Mexico; Faculty of Chemistry, National Autonomous University of Mexico, Mexico City, 04510 Mexico

**Keywords:** Acacia pods, Antioxidant activity, Damage protection, Free radicals

## Abstract

**Background:**

Obesity is a worldwide public health issue, reaching epidemic condition in developing countries associated to chronic diseases. Oxidative damage is another side effect of obesity. Antioxidant activity from plant components regulates at some extent this imbalance. Main goal of the present study was to determine the antioxidant activity and protection against oxidative-induced damage of *Acacia shaffneri* (AS) and *Acacia farnesiana* (AF) pods extracts.

**Methods:**

To evaluated antioxidant activity and radical scavenging capacity of AS and AF extracts, two experiments were performed: 1) pods extracts were challenged against H_2_O_2_ using kidney cells in an in vitro assay; and 2) (*Meriones unguiculatus*) was employed in an in vivo assay to observe the effect of pods extracts on scavenging properties in plasma.

**Results:**

Both pods extracts presented an important protective effect on radical scavenging capacity against ABTS• + and DPPH^+^, and also in TBARS formation in vitro. Vegetal pods extracts did not induce any pro-oxidative effect when added to kidney cells in DMEM. Cells damage in DMEM with addition of H_2_O_2_ was significantly higher than those when vegetal pods extracts were added at 50 (P < 0.05) or 200 ppm (P < 0.001). Plasma scavenging properties presented an important dose-dependent positive effect in those groups where pods extracts were administered.

**Conclusions:**

The antioxidant protection of the acacia pods extracts reported in this study suggests the possible transference of antioxidant components and protective effects to animal products (milk, meat, and by-products) from Acacia pods when this vegetation is included in the diet. In order to evaluate, the possible transference of theirs antioxidant components to animal products, the incorporation of these non-conventional resources to ruminant feeding is a good opportunity of study. Profiling of *Acacia* farnesiana pods extract is necessary to identify the responsible bioactive compounds of protective properties.

## Background

Obesity is a worldwide public health issue, reaching epidemic condition in developing countries. Mexico has a 32.8 % of adult obesity associated to malnutrition, increasing the incidence of hearth stroke, type 2 diabetes, cancer, premature death and inflammation [1]. Oxidative damage is another side effect of obesity, this physiological status occurs when endogenous system loses balance. Antioxidant activity from plant components regulates at some extent this imbalance by increasing the scavenging activity of reactive oxygen species (ROS) such as hydrogen peroxide (H_2_O_2_). The inicial steps of development of degenerative diseases are preventing by protection of target molecules as lipids, proteins and nucleic acids from oxidative damage [2]. Many plants distributed in arid and semi-arid environments have alternative uses such as forage resources; traditional medicine and more recently, they have been pointed out as antioxidant sources [3–5], mainly due to their polyphenol content. These compounds have received attention because of their antioxidant capabilities and their protection against ROS-induced damage [2, 6] with biological functions like antiinflammatory, anticancer, antiobesity, antiaging [7], hepatoprotective and antidiabetic properties [8]. Acacia species are widely distributed in arid, semiarid and tropical regions of Mexico and all around the world. The extracts obtained from these plants have previously shown antimicrobial [3], antihelmintic [9] and antioxidant [4] properties.

Information of *Acacia* in terms of its antioxidant capacity is very limited; however Hannachi et al. [10] reported the protective response against 2,2′-azino-bis (3-ethylbenzthiazoline-6-sulphonic acid) radical cation (ABTS•+) and 1,1-diphenyl-2-picrylhydrazyl (DPPH^+^) radicals (0.78 and 2.4 mM trolox equivalents, respectively). They found a polyphenol content of 276 mg gallic acid equivalents/100 g dry matter and identified the flavonoids gallic acid, methyl gallate, myricetin, naringenin, and quercetin in pods of this species.

Acacias have been found as a natural forage resource in these marginal regions. Improving the utilization of local vegetation for livestock production would let to increase the functional quality of animal products (milk, meat, and by-products), supporting a healthy and appropriate diet for human while promoting preventive actions aimed to reduce chronic maladies related to obesity.

The main goal of the present study was to determine the antioxidant activity and protection against oxidative-induced damage of crude extracts of *Acacia shaffneri* and *Acacia farnesiana* pods. Secondly, we simulated the potential preventive influence of antioxidant compounds. Two experiments were performed: 1) the protective effect of crude extracts of *Acacia* pods were challenged against H_2_O_2_ using pig kidney LLC-PK1 cells in an in vitro assay; and 2) gerbils (*Meriones unguiculatus*) were employed in an in vivo model to observe the effect of crude extract of *Acacia* pods on the plasma antioxidant capacity.

## Methods

### Reagents

2,2′-azobis(2-amidinopropane dihydrochloride (AAPH; Sigma-Aldrich St. Louis, MO. USA. cat. 440914). Trolox (6–Hydroxy–2,5,7,8–tetramethylchroman–2–carboxylic acid; Calbiochem, Billerica, Massachusetts. U.S. cat. 648471). Fluorescein (Sigma-Aldrich St. Louis, MO. USA. cat. F6377). Dulbecco’s Medium Modified (DMEM; Gibco BRL. Waltham, MA, USA. cat. 12800-017). Tryple Express (Gibco BRL Waltham, MA, USA. cat. 12604013). Fetal bovine serum (Gibco BRL Waltham, MA, USA. cat. 16000-044). Penicillin/streptomycin (Gibco BRL Waltham, MA, USA. cat. 15140122). H_2_O_2_ (J.T. Baker Center Valley PA, USA. cat. 2186). 5(6)-carboxy-2′,7′-dichlorodihydrofluorescein diacetate (carboxy-H_2_DCFDA) (Molecular Probes Waltham, MA, USA. cat. C-400).

### Vegetal extracts obtention

Vegetal samples evaluated in this study were collected in February of 2014 in the semiarid region of central Mexico at 20° 35′ latitude North and 100°18′ longitude West within the Queretaro state. The place is 1950 masl with 460 mm of average precipitation per year. The person responsible for the botanical identification was Manuel Rivera, a staff member of the FES-Cuautitlan herbarium at the National Autonomous University of Mexico (UNAM). The internal identification number of the material is 8757 for *A. farnesiana* and 8765 for *A. schaffneri. A. farnesiana* and *A. schaffneri* pods were collected, managed and extracted (methanol:water, 80:20 v/v) to obtain a crude extract, as described by Cuchillo et al. [3]. Total polyphenols were determined with the recommendations of the same authors.

### Antioxidant activity in vitro

Free radical scavenging capacity of crude extracts of AF and AS pods was evaluated using the decoloration reduction of ABTS• + assay from Re et al. [11]. An appropriate solvent blank reading was taken (*A*_B_). After the addition of 100 μL of ethanol extracts solution (200 ppm) to 3 ml of ABTS• + solution, the absorbance reading was taken at 30 °C for 10 min after initial mixing (*A*_E_). All solutions were used on the day of preparation. The percentage of inhibition of ABTS• + was calculated using the following formula: % scavenging activity = [(*A*_B_-*A*_E_)/*A*_B_] × 100. Where *A*_B_ = absorbance of the blank, and *A*_E_ = absorbance of the crude extracts. Catechin and α-tocopherol standards (100 ppm) were used as references.

Another assay to evaluate the antioxidant scavenging capacity for crude extracts of pods was done using DPPH^+^ assay according to Von Gadow et al. [12] with some modifications. Briefly, 0.25 ml of extract solution (200 ppm), were added to 2 ml 0.36 mM DPPH^●^ solution. The mixture was shaken vigorously and left stand for 30 min in the dark. The absorbance was measured at 513 nm at *t* = 0 and after 30 min using a Beckman DU-70 spectrophotometer. Radical scavenging activity was calculated according to the same equation used above. Butylated hydroxyanisole (BHA) and α-tocopherol standards (100 ppm) were used as references.

Liposomes were prepared according to Tsuda et al. [13]. Briefly, soy lecithin (10 g) was dispersed in a sodium phosphate buffer (100 ml, 20 mM, pH 7.4) and sonicated (Bioblock Scientific, Vibra cell, Newton, CT; USA) for 2.5 h in an ice-cold water bath. Crude extracts of AF and AS pods were tested for lipid peroxidation activity with the following mixture: extract (0.5 ml, 200 ppm), liposomes (2 ml), 25 mM FeCl_3_ (0.1 ml), 25 mM H_2_O_2_ (0.1 ml), 25 mM ascorbic acid (0.1 ml) and 0.2 M phosphate buffer (1.2 ml, pH 7.4). Mixture was incubated at 37 °C/4 h and 1 ml of BHA (20 mg/ml in methanol) was added to stop the reaction. Oxidation of liposomes was subsequently determined by measuring the thiobarbituric acid-reactive substances (TBARS) by adding thiobarbituric acid and HCl (1 and 10 %, respectively, 1 ml each) and heating the mixture (100 °C/30 min). Mixture was cooled in an ice bath (15 min) and 5 ml of chloroform (J.T. Baker, 99.8 %) were added, then it was centrifuged (3,000 × g/20 min). Absorbance of the supernatant was measured at 532 nm (Beckman DU-70 spectrophotometer). Control was prepared by adding ethanol instead of vegetal extract, and a blank without liposomes but methanol was also analyzed. Inhibition of TBARS formation (%) was calculated with the following equation: 100 × (*A*_0_ − *A*_t_)/(*A*_0_ − *A*_1_) where *A*_0_, *A*_1_, and *A*_t_ are values for control, blank, and samples absorbance, respectively.

To evaluate the antioxidant activity in cells in culture, pig kidney cells LLC-PK1 (Lilly Laboratory Culture Porcine Kidney Type 1, obtained from American Type Culture Collection (Rockville, MD, USA) were employed. Cells were incubated in 48 well plates for 24 h under the following conditions: 37 °C, 5 % CO_2_ / 95 % atmospheric air (NUAIRE-5820 incubator) in DMEM with 10 % FBS for 24 h. Then DMEM with FBS was replaced with 250 μL of DMEM, and the incubation was set for another 30 min, under the same conditions. Vegetal extracts solutions were added (5 μL of 2,500 or 10,000 ppm) to get final concentrations of 50 or 200 ppm in half of wells, respectively, and plates were incubated for 30 additional minutes. An H_2_O_2_ solution (12.5 μL, 20 mM) was used as external oxidant; and it was added only to half of wells of each vegetal extract concentration in order to detect a possible pro-oxidant effect; and they were incubated for 2 h. After discarding DMEM, all wells were washed with 250 μL of phosphate buffer solution (PBS) at 37 °C. After discarding it, a 6-carboxy-2′,7′-dichlorodihydrofluorescein diacetate (carboxy-H_2_DCFDA) solution was added (250 μL, 15 μM in PBS). A final incubation of 20 min took place before replacing carboxy-H2DCFDA solution by cold DMEM (250 μL, 4 °C). All volumes mentioned were considered for each well and incubation conditions were always the same. Cells were observed to detect ROS presence in cytoplasm using an inverted microscope (Nikon Eclipse TS 100) under minimum illumination conditions with a regular lamp to detect cells and then, using different filters (488 nm excitation and 530 nm emission for carboxy-DCF) to visualize fluorescence. Emission camps (brilliant green) were photographed and quantified using a NIS-Elements software (2.3 and 3.0, respectively) according to Hernández-Fonseca et al. [14].

### Antioxidant activity in vivo

A total of 72 Mongolian gerbils (*Meriones unguiculatus*) males and females, pathogen-free, 10-18 weeks old, weight between 65-89 g originally purchased from Charles River Laboratories (Wilmington, MA, USA) were used and housed in polypropylene cages on hard wood chip bedding in groups of five-eight/cage. Food and water were provided *ad libitum* in a temperature-controlled room (22 ± 1 °C) with a 12-h light–dark cycle, and 40-60 % humidity in the specific pathogen-free animal facility at National Institute of Medical Science and Nutrition Salvador Zubirán.

Animals’ adaptation period included physical contact twice a week during bedding change and weekly when weight was registered from two weeks before dosage onset to the end of the study. Protocols for maintenance, sample collections and euthanasia were reviewed and approved by the Institutional Animal Care and Research Advisory Committee (Comité de Investigación en Animales-CINVA).

Animals were randomly divided as follows: three doses groups (8, 16 and 32 mg of total polyphenols (TP) /100 g body weight); one positive control group (0.5 mg ascorbic acid/100 g body weight); and one negative control group (acidified water, HCl 50 ppm, pH 2.4). Calculations were made in order to give 1.0 mL/100 g body weight. The dose was given orally every day at 8:00 am using oral gavage needles made of stainless steel with a rounded-silver tip adjusted in a 1.0 mL syringe.

### Biochemical determinations in plasma

In order to collect blood samples, an hour after last dosage, animals were anesthetized intraperitoneally with a ketamine/xylacine mix (7.0/0.8 mg/100 g weight, respectively). Once ocular and patellar reflexes were gone, cardiac puncture was done in a hair-free thoracic area to get at least 1 mL of blood in a Vacutainer blood collection tube with EDTA as anticoagulant.

DPPH^+^ scavenging capacity was determined according to Koren et al. [15]: once plasma was obtained, two reaction tubes per sample were prepared with 25 μL of saline, 25 μL of plasma, and 50 μL of DPPH^+^ solution (1 mmol/L, light-protected). Blanks were prepared replacing plasma with 25 μL of Hank’s balance salt solution. All tubes were incubated for 2 min at room temperature in darkness, and 800 μL of absolute ethanol were added, they were inverted few times for homogenization, and incubated for 2 additional minutes in same conditions. Tubes were centrifuged at 15,000 × g for 2 min, absorbance of supernatant was measured at 517 nm. Results were such as percent (%) of DPPH^+^ scavenged, calculated by the following formula: [(optical density of control-optical density of compound)/(optical density of control)*100]. Ascorbic acid was used as a reference compound [15].

Oxygen radical absorbance capacity (ORAC) assays were performed in a Synergy™ HT Multi-Mode Microplate Reader (BioTek Instruments, Inc., Winooski, VT, USA) and were based on the method described by Huang et al. [16]. In these assays 2,2′-azobis-(2-amidinopropane dihydrochloride) (AAPH), a water-soluble azo compound, was used as a peroxyl radical generator; Trolox, a water-soluble vitamin E analogue, was used as standard. Briefly, 25 uL of water, Trolox standards and diluted samples were mixed with 25 uL of 153 mM AAPH and with 150 uL of 50 nM fluorescein and incubated at 37 °C. The fluorescence was measured every minute for 90 min using fluorescence filters for an excitation wavelength of 485 nm and an emission wavelength of 520 nm. The ORAC values were calculated using the net area under the decay curves.

### Statistical analyses

A descriptive analysis was done to know response distribution. An ANOVA analysis was used as non-parametric test using Kruskal-Wallis for all groups and U de Mann-Whitney for peers to detect differences among groups and doses with SPSS statistic software (18^th^ version, IBM Corporation, New York, USA). Differences were considered significant when P ≤ 0.05.

## Results

### In vitro assays

#### Total polyphenol content, antioxidant activity of crude extract of *Acacia shaffneri* and *Acacia farnesiana* pods

Total polyphenol content in *A. farnesiana* and *A. schaffneri* was 76 and 213 equivalents of gallic acid/g of extract. In Table 1, we observe that both pod extracts presented an important protective effect on radical scavenging capacity against ABTS• + and DPPH^+^, and also in TBARS formation. On ABTS• + radical scavenging capacity no significant difference was found between extracts (*P* > 0.05). However, both extracts were significantly less effective (P < 0.05) than alpha-tocopherol and catechin standards. When extracts were tested using DPPH^+^ no significant difference (*P* > 0.05) was found between them; neither when compared to alpha-tocopherol nor BHA standards. In relation to inhibition of TBARS formation in liposomes, extracts did show significant differences (*P* > 0.05) between them, but they were significantly different (P < 0.05) when compared to alpha-tocopherol standard (Table 1).

**Table 1:** In vitro antioxidant activity (%) of crude extracts from *Acacia shaffneri* (AS) and *Acacia farnesiana* (AF) pods

Free radicals	AS	AF	alpha-tocopherol	catechin	BHA
ABTS•+	10.85^b^ ± 0.08	10.47^b^ ± 0.36	92.64^a^ ± 0.51	91.99^a^ ± 1.00	ND
DPPH^●^	94.77^a^ ± 0.36	95.18^a^ ± 0.26	93.42^a^ ± 0.69	ND	92.36^a^ ± 0.25
TBARS	66.59^b^ ± 1.23	66.05^b^ ± 1.62	92.54^a^ ± 0.72	ND	ND

*ABTS• +* 2,2′-azino-bis (3-ethylbenzthiazoline-6-sulphonic acid; *DPPH* ^●^ 1,1-diphenyl-2-picrylhydrazyl; *TBARS* thiobarbituric acid reactive substances, *BHA* buthylated hydroxyanisole; *ND* not determined

^a,b^ Means with different letters within the same row are significantly different at P < 0.05

**Fig. 1:**
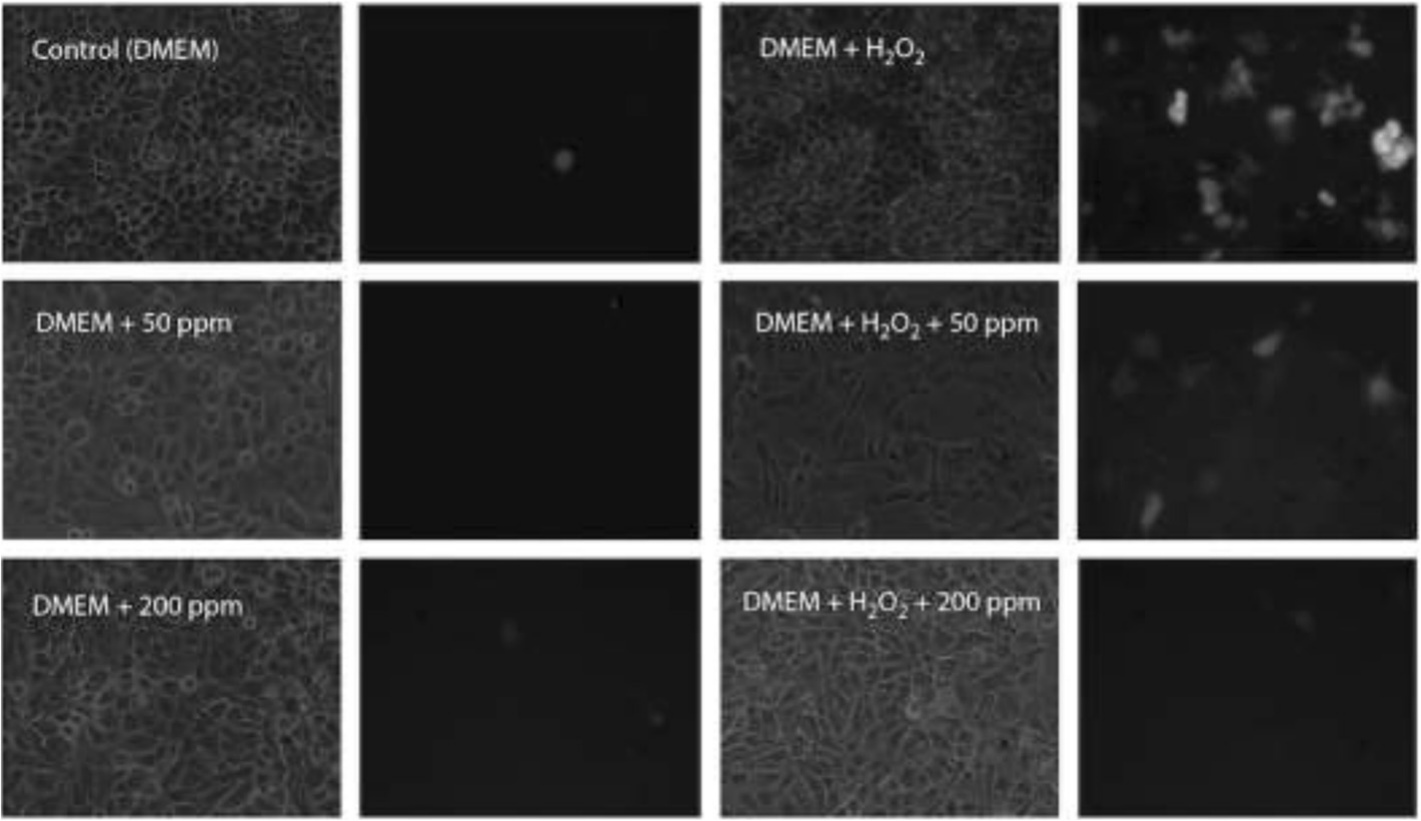
Effect of 50 and 200 ppm of crude extract of *Acacia shaffneri* pods in porcine kidney cells cultivated in Dulbecco’s modified eagle medium (DMEM) with or without H_2_O_2_

**Fig. 2:**
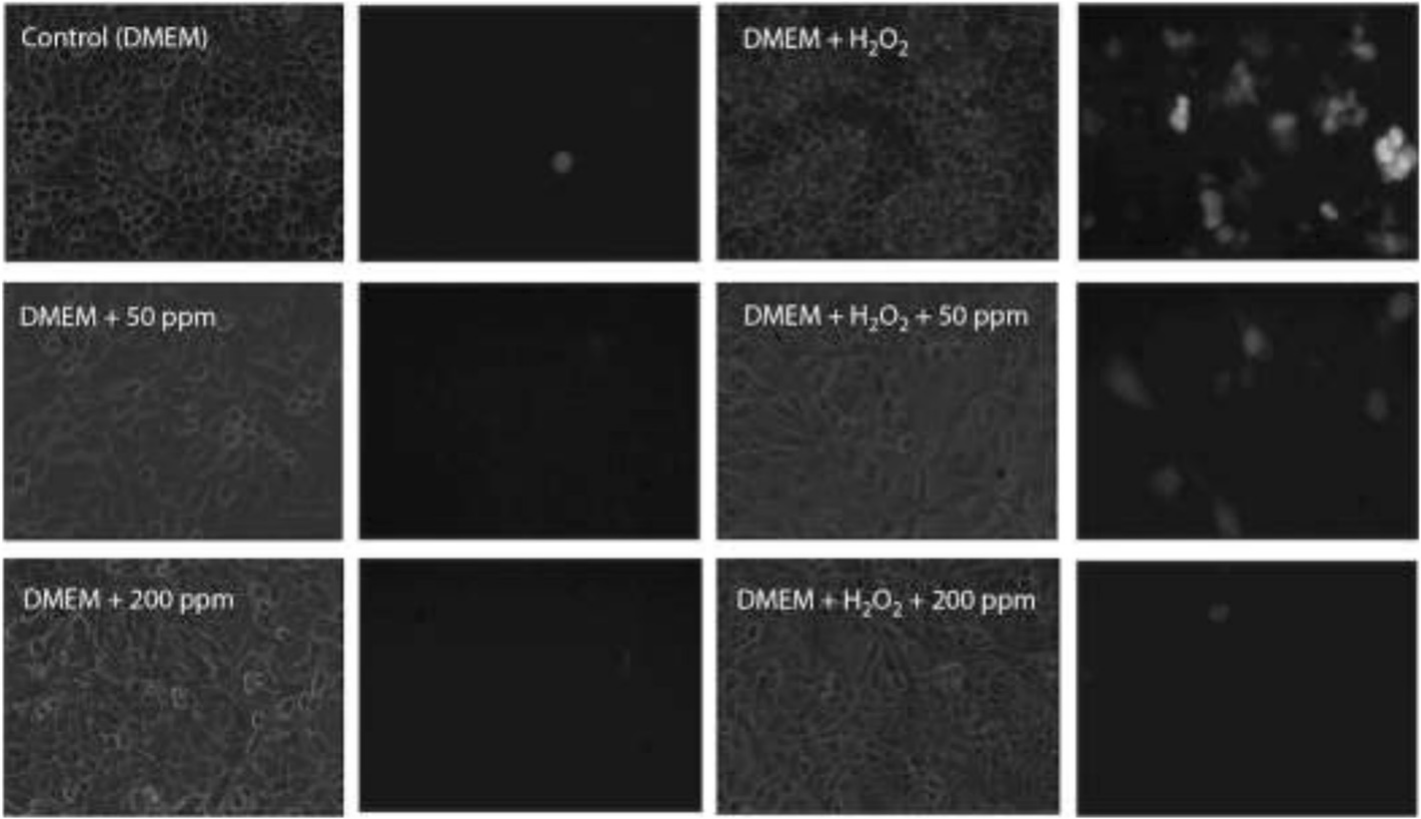
Effect of 50 and 200 ppm of crude extract of *Acacia farnesiana* pods in porcine kidney cells cultivated in Dulbecco’s modified eagle medium (DMEM) with or without H_2_O

**Fig. 3:**
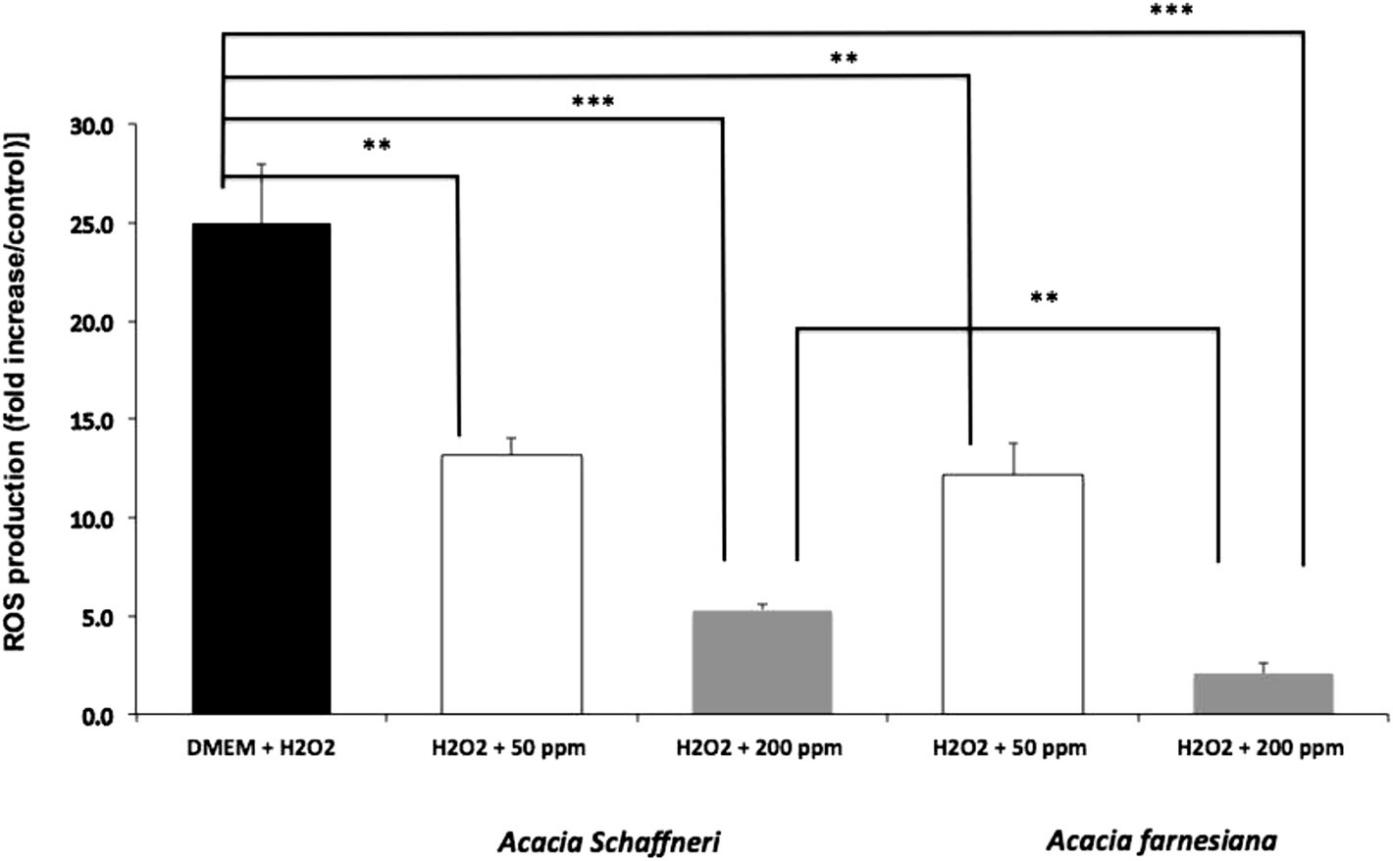
Quantitative protection of crude extracts of *Acacias* pods in porcine kidney cells against H_2_O_2_-oxidative-induced damage at two different concentrations

According to the statistical assessment of the potential protection against oxidative-induced damage on kidney cells of crude extracts AS and AF pods, by a Kruskal-Wallis test and the confirmatory U de Mann Whitney, we found that were not significant differences (*P* > 0.05) among extracts at 50 ppm concentration and control. However, at 200 ppm concentration of crude extract of AS and AF pods there were significant differences (P < 0.05) between extracts in relation to H_2_O_2_ and control cells, being crude extract of AF pods which presented the highest antioxidant protection capacity (Figs. 1, 2, and 3). We can also suggest that crude extracts of AS and AF pods at 200 ppm concentration have no pro-oxidant effect since no cell damage was observed and the fluorescence signal was similar to those of the kidney cells in DMEM alone.

#### Protection against oxidative-induced damage in porcine kidney cells of crude extracts of *Acacia shaffneri* and *Acacia farnesiana* pods

As shown in Fig. 1, AS crude extract presented a protective effect of kidney cells against H_2_O_2_-induced damage; either with 50 or 200 ppm concentrations. These results are clearly different from those obtained from DMEM alone or with H_2_O_2_, where cells lysis is certainly observed as brilliant green fluorescent spots. We also found that crude extrac of AS pods did not have pro-oxidant effect at the concentrations evaluated in this study. From Fig. 2, we observed that crude extract of AF pods presents similar antioxidant protective effect when defied against H_2_O_2_ as oxidative aggressor of kidney cells.

Figure 3 allows comparing this protective effect in a quantitative basis. Previously, we made sure that crude extract of both species pods did not induce any pro-oxidative effect on porcine kidney cells at none concentration evaluated in the present study. We can state that cells damage in DMEM with addition of H_2_O_2_ is significantly higher than those when crude extracts were added either at 50 (P < 0.05) or 200 ppm (P < 0.001). In relation to AS compared to AF when added at 50 ppm there was no difference (*P* > 0.05) between species when H_2_O_2_ was added as aggressor factor. However, at 200 ppm AF showed a better protective effect than AS (P < 0.05).

### In vivo assay

#### DPPH^+^ scavenging capacity and ORAC of gerbil plasma with three different dosages of crude extract of *Acacia shaffneri* and *Acacia farnesiana* pods

Table 2 shows the effect of different concentrations of crude extracts of pods on DPPH^+^ scavenging capacity and ORAC. As expected the highest concentration (32 mg/100 g BW) presented the best effect of both crude extracts (AF and AS) on DPPH^+^ and ORAC. As extracts of 16 and 32 mg/100 g BW doses were no significantly different (*P* > 0.05) from positive control (C+, ascorbic acid), it is important to point out that a lower concentration of AF and AS showed higher scavenging capacity than dose from basal and negative control (C-, acidified water) groups on both assays. For ORAC results, AS extracts (8, 16, and 32 mg/100 g BW) showed the highest values among all groups, even when compared to C+ group. On the other hand, when AF was administered in a concentration of 32 mg/100 g BW, presented no significant difference (*P* > 0.05) from C+ group and lower concentrations of AF showed higher values when compared to basal group.

**Table 2:** Effect of different concentrations (8, 16 and 32 mg TP/100 g BW) of crude extracts of *Acacia farnesiana* (AF) and *Acacia shaffneri* (AS) pods on DPPH^●^ scavenging capacity (%) and oxygen radical absorbance capacity (ORAC) (μM Trolox equivalents/L) of gerbil (*Meriones unguiculatus*) plasma

Vegetal extract	AF			AS			Basal	* C-	** C+
	8	16	32	8	16	32			
DPPH^●^	21.8^d^ ± 2.4	20.0^e^ ± 1.7	30.0^a^ ± 2.0	22.2^d^ ± 3.4	26.2^c^ ± 2.1	29.0^b^ ± 1.7	14.9^f^ ± 2.4	14.7^f^ ± 2.4	26.1^c^ ± 1.6
ORAC	1339^d^ ± 246	1309^d^ ± 102	1561^c^ ± 156	2390^a^ ± 569	2245^a^ ± 550	2207^a^ ± 441	1090^e^ ± 88	1213^d^ ± 166	1888^b^ ± 223

*BW* body weight; *DPPH* ^●^ 1,1-diphenyl-2-picrylhydrazyl radical

*C- = negative control (acidified water)

** C+ = Positive control (ascorbic acid, 0.5 mg/100 g BW)

^a,b,c,d,e,f^ Means with different letters within the same row are significantly different at P < 0.05

## Discussion

Obesity condition has multiple side effects incluiding cell oxidative damage resulting in many cases in chronic maladies. Such diseases could be preventible at some extent by hindering the initial process from oxidative damage caused by reactive oxygen species [2]. Some of the compounds being part of the phytochemical group responsible for these desirable effects are the polyphenols. Total polyphenol content in the present study was in accordance with the results reported previously by Singh et al. [17] with values ranging from 19 to 657 mg of gallic acid equivalents/g of extract whereas Singh et al. [18] reported values for *A. auriculiformis* from 300, 495 and 775 mg of gallic acid equivalents/g in crude extract, ethyl acetate and water fractions, respectively. *Acacia* genus is recognized as a rich source of phytochemicals however, *Acacia* profiling has not been subject to complete study. To our knowledge, few studies have investigated extensively in this respect. Therefore it is recommended to identify bioactive compounds in further investigations of the extracts to isolate, to purify and to test them to suggest specific applications and/or recommendations.

*Acacia* pod extracts showed a protective effect on radical scavenging capacity against ABTS•+, DPPH^+^, and TBARS formation in vitro. A previous study had reported a functional bioactive compound in *A. farnesiana* [3] as methyl gallate and Crispo et al. [6] suggested that methyl gallate can significantly attenuate the apoptotic response as a result of a long-term oxidative stress, atenuanting this process throught direct and indirect scavenging of reactive oxygen species (ROS). Our results on crude pod extracts did not induce pro-oxidative effects when added to porcine kidney cells in DMEM. Cells damage in DMEM with addition of H_2_O_2_ was significantly lower when pod extracts were added at 200 ppm than when added at 50 ppm. Maldini et al. [19] reported the presence of strong antioxidant polyphenols constituents in *A. nilotica* pods which were evaluated employing trolox equivalent antioxidant capacity (TEAC) assay; where a strong free radical scavenging activity on ABTS• + cation was showed. The presence of different derived galloylated of catechin and gallocatechin are compounds with antioxidant properties. Some of these compounds recently reported in the genus *Acacia* as gallic acid methyl ester-4-gallate, gallocatechin-7,3′-digallate, gallocatechin-7,4′-digallate and 1,3-di-*O*-galloyl-ß-D-glucopyranose, could be further found in our extracts. It is important to notice that higher polyphenol content from *A. shaffneri* did not was related to higher protection against the oxidative stress damage by H_2_O_2_ since porcine kidney cells showed a lower oxidative damage when crude extact of *A. farnesiana* was added. This suggests that some other compounds that were not considered in this study could be responsible for such radical scavenging activity. Ramli et al. [20] evaluated the antioxidant activity of ethanolic extract of *A. farnesiana* leaves, on scavenging DPPH^+^ radical, showing a 80 % of activity with 100 μg/ml, which is lower than the 95.2 % obtained in the present study, with a concentration of an methanol:water extract of 200 μg/ml in pods. On the other hand, previous unpublished assays in leaves (AF) and whole plant (AS; leaves, stems and pods) MeOH:H_2_O (80:20 v/v) extracts, with same concentration (200 μg/ml) showed a DPPH^+^ radical scavenging activity of 95.1 and 94.8 %, respectively. We reported [4] an important polyphenol content in *A. farnesiana* (AF) complete plant (38,170 mg gallic acid equivalents/kg dry matter). Many other compounds in AF have been reported such as flavonoids (12 mg rutin equivalents/100 g dry matter), carotenoids, coronaric, oleic, linoleic and linolenic acids, and some aldehydes (geraniol and geranyl acetate) [3, 6, 21].

The oral administration of rich-polyphenol extracts increased plasma free radical scavenging properties and presented an important dose-dependent positive effect in those groups where Acacia pod extracts were dispensed. Haruenkit et al. [22] reported the protective antioxidant effect of natural products from exotic tropical fruits in plasma rats fed with 1 and/or 5 % of durian, snake and mangosteen fruits, showed a similar antioxidant activity (28.1, 21.1 and 10.5 %, respectively) when compared to antioxidant activity from gerbil plasma which were dosified with 8, 16 and 32 mg of total polyphenols/100 g of BW with extract pods from *A. farnesiana* (21.8, 20.0 and 30.0 %) and from *A. Schaffneri* (22.2, 26.2 and 29.0 %, respectively). This response was likely related to the phytochemicals compounds mainly polyphenols [2, 19] as ferulic acid and its metabolites can increase radical scavenging properties of plasma in rats after the ingestion of this compound in the diet [23, 24]. Nevertheless, supra-physiological concentrations of certain polyphenols can potentially interfere with many disease-related biochemical processes acting as pro-oxidants [6]. Both species evaluated in the present study showed resemble antioxidant activity in vitro whereas AF presented the best protective capacity in vivo. However, larger abundance of *Acacia* and wide distribution of AF along the Mexican territory, suggests that this species could be a more important source of bioactive compounds than AS.

## Conclusions

The antioxidant protection of the acacia pods extracts reported in this study suggests the possible transference of antioxidant components and protective effects to animal products (milk, meat, and by-products) from Acacia pods. The incorporation of these non-conventional resources in ruminant feeding should be further evaluated. Profiling of *Acacia* farnesiana pods extract is necessary to identify responsible bioactive compounds of the showed antioxidant and protective properties.
